# Integrated analysis of lncRNA, miRNA and mRNA profiles reveals potential lncRNA functions during early HIV infection

**DOI:** 10.1186/s12967-021-02802-9

**Published:** 2021-04-01

**Authors:** Lianwei Ma, Hui Zhang, Yue Zhang, Hailong Li, Minghui An, Bin Zhao, Haibo Ding, Junjie Xu, Hong Shang, Xiaoxu Han

**Affiliations:** 1 NHC Key Laboratory of AIDS Immunology (China Medical University), National Clinical Research Center for Laboratory Medicine, The First Affiliated Hospital of China Medical University, No 155, Nanjing North Street, Heping District, Shenyang, 110001 Liaoning China; 2 Key Laboratory of AIDS Immunology, Chinese Academy of Medical Sciences, Shenyang, 110001 China; 3 Key Laboratory of AIDS Immunology of Liaoning Province, Shenyang, 110001 China; 4 Collaborative Innovation Center for Diagnosis and Treatment of Infectious Diseases, 79 Qingchun Street, Hangzhou, 310003 China

**Keywords:** HIV-1, lncRNA, mRNA, miRNA, *Cis*-regulatory, ceRNA

## Abstract

**Background:**

Long noncoding RNAs (lncRNAs) can regulate gene expression in a *cis*-regulatory fashion or as “microRNA sponges”. However, the expression and functions of lncRNAs during early human immunodeficiency virus (HIV) infection (EHI) remain unclear.

**Methods:**

3 HAART-naive EHI patients and 3 healthy controls (HCs) were recruited in this study to perform RNA sequencing and microRNA (miRNA) sequencing. The expression profiles of lncRNAs, mRNAs and miRNAs were obtained, and the potential roles of lncRNAs were analysed based on discovering lncRNA *cis*-regulatory target mRNAs and constructing lncRNA–miRNA–mRNA competing endogenous RNA (ceRNA) networks. Then, Gene Ontology (GO) and Kyoto Encyclopedia of Genes and Genomes (KEGG) pathway enrichment analyses were performed on 175 lncRNA-associated differentially expressed (DE) mRNAs to investigate the potential functions of DE lncRNAs in ceRNA networks.

**Results:**

A total of 242 lncRNAs, 1240 mRNAs and 21 mature known miRNAs were determined as differentially expressed genes in HAART-naive EHI patients compared to HCs. Among DE lncRNAs, 44 lncRNAs were predicted to overlap with 41 target mRNAs, and 107 lncRNAs might regulate their nearby DE mRNAs. Two DE lncRNAs might regulate their *cis*-regulatory target mRNAs *BTLA* and *ZAP70,* respectively, which were associated with immune activation. In addition, the ceRNA networks comprised 160 DE lncRNAs, 21 DE miRNAs and 175 DE mRNAs. Seventeen DE lncRNAs were predicted to regulate *HIF1A* and *TCF7L2,* which are involved in the process of HIV-1 replication. Twenty DE lncRNAs might share miRNA response elements (MREs) with *FOS*, *FOSB* and *JUN,* which are associated with both immune activation and HIV-1 replication.

**Conclusions:**

This study revealed that lncRNAs might play a critical role in HIV-1 replication and immune activation during EHI. These novel findings are helpful for understanding of the pathogenesis of HIV infection and provide new insights into antiviral therapy.

**Supplementary Information:**

The online version contains supplementary material available at 10.1186/s12967-021-02802-9.

## Introduction

Early human immunodeficiency virus infection (EHI) reflects the period following viral entry during which viremia bursts occur until decreasing to a stable viral load level approximately 6 months post infection [1–5]. During EHI, viremia increases exponentially, while proinflammatory cytokines are produced by innate immune cells and coincide with mounting adaptive immune responses. Meanwhile, the viral reservoir is formed early in infection. It has been reported that the events occurring during EHI are critical in determining the transmission rates, the course of disease progression, and HIV-related morbidity and mortality [6]. To date, the pathogenesis of EHI remains unclear. Therefore, exploring the mechanism of HIV pathogenesis during EHI is helpful for the design of therapeutic strategies and vaccine development.

Long noncoding RNAs (lncRNAs), which are longer than 200 nt and do not encode proteins, have attracted much attention in recent years [7, 8]. LncRNAs can act as guides, scaffolds, decoys, signalling molecules or via genomic targeting, acting as *cis-*or *trans-*regulatory elements or through antisense interference to play vital roles in a number of biological processes [9–12]. Moreover, lncRNAs bind to specific miRNAs and regulate their functions by acting as “miRNA sponges”, which are known as competing endogenous RNAs (ceRNAs) [13, 14]. For example, lncRNA MT1JP regulates the expression of *FBXW7* by competitively binding to miR-92a-3p, which in turn affects the progression of gastric cancer [15]. Therefore, it will be of interest to explore the functional roles of lncRNAs in different research fields.

Several HIV-related transcriptome analyses have focused on the potential functions of lncRNAs, such as the differential expression of lncRNAs during HIV-1 and HIV-2 infection [16], the impact of lncRNAs on HIV replication [17], the roles of lncRNAs in the establishment and reversal of latent HIV infection [18, 19] and the relationships between lncRNAs and disease progression [20]. For example, lnc-RNF125-1, lnc-TRAF5-1 and lnc-TRDMT1-1 might control HIV replication by regulating the expression and function of nearby mRNAs in a *cis*-regulatory fashion [17]. Furthermore, 3 lncRNAs, PVT1, RP11347C18.3, and RP11-539 L10.2, are dysregulated genes in HIV latency and might be targets for HIV latency reversal [19]. However, the potential roles of lncRNAs during EHI have not been systematically investigated.

In this study, we analysed the lncRNA, miRNA, and mRNA expression profiles from 3 HAART-naive EHI patients and 3 healthy controls (HCs) using RNA sequencing (RNA-Seq) and microRNA (miRNA) sequencing (miRNA-Seq). The potential roles of lncRNAs were analysed based on the prediction of lncRNA *cis*-regulatory target mRNAs and by constructing lncRNA–miRNA–mRNA ceRNA networks. We found several DE lncRNAs might involve in HIV-1 replication and immune activation during EHI. Our novel findings will benefit further exploration of the pathogenesis of HIV and propose new potential targets for therapeutic strategies.

## Materials and methods

### Ethics

This study was approved by the Medical Research Ethics Committee of the First Affiliated Hospital of China Medical University (Shenyang, China). All participants provided written informed consent for participation in this study.

### Patients

The HAART-naive EHI patients in our study were recruited from a large-scale, prospective high-risk men who had sex with men (MSM) cohort in Liaoning, China. In this cohort, HIV-1-negative MSM were followed up every 8 weeks. A fourth-generation enzyme-linked immunosorbent assay (ELISA) was used to detect HIV-1 infection status, and western blotting was applied for further validation. HIV-1 RNA amplification testing was performed on ELISA-negative samples. HIV infection time was estimated as the previous 14 days from the date on which the sample testing result was RNA-positive and ELISA-negative or the midpoint between the last negative and first positive results of the ELISA screening tests [21]. The stage within 180 days post HIV infection was defined as EHI.

Three HAART-naive EHI patients and 3 HCs were enrolled for RNA-seq and miRNA-seq. For the 3 EHI patients, the level of CD4^+^ T cell counts and viral loads (VLs) were similar. The 3 EHI patients and 3 HCs were recruited from the same high-risk MSM cohort mentioned above and all participants were males with similar ages and none of them were infected by herpes simplex virus (HSV), syphilis, hepatitis B virus (HBV) or hepatitis C virus (HCV). Also, none of them were  in use of alcohol, recreational drugs or nicotine products. The clinical characteristics of subjects were listed in Additional file 1: Table S1. In addition, we performed RT-qPCR validation on 10 EHI patients and 7 HCs, whom were recruited from the same cohort as mentioned above. The age, lifestyle and co-morbidities status were no statistically different between EHI patients and HCs (Additional file 1: Table S1).

Peripheral blood mononuclear cells (PBMCs) from EHI patients and HCs were isolated by density gradient centrifugation with Ficoll–Hypaque (GE Healthcare), cryopreserved in foetal calf serum (Gibco) supplemented with 10% DMSO (Sigma) and stored in liquid nitrogen.

### Laboratory testing

Anticoagulated whole blood of HIV-1 infected patients was collected at each follow-up visit after they were diagnosed as HIV-1 infection. The CD4^+^T cell count was determined using FACS Calibur™ flow cytometer (Becton–Dickinson) and plasma viral load (VL) was detected by the COBAS AmpliPrep/COBAS TaqMan HIV-1 test (Roche). A Syphilis screening serological test was performed with the Rapid Plasma Reagin (RPR) test (Shanghai Kehua Bio-engineering Co). The Treponema Pallidum particle (TPPA) assay (Serodia TPPA) was used to confirm the positive RPR results. Subjects with both the TPPA and RPR plasma positive were considered as current infection. Herpes simplex virus type 2 (HSV-2)-specific immunoglobulin G (IgG) antibody testing was performed to detect HSV-2 infection using ELISA methods (HerpeSelect-2 ELISA IgG; Focus Diagnostics). Wondfo HIV-HCV-TP-HBsAg Multi-Test Kits (Colloidal Gold) were used to detect HBV and HCV infection.

### RNA isolation and quality control

Total RNA was isolated from PBMCs of 3 HAART-naive EHI patients and 3 HCs using TRIzol reagent (Life Technologies). TurboDnase (Life Technologies) was used to eliminate DNA. RNA concentrations were measured with a NanoDrop ND-2000 instrument (Thermo Fisher Scientific), and RNA quality was estimated by denaturing agarose gel electrophoresis for subsequent sequencing. A NanoDrop ND-1000 instrument (Thermo Fisher Scientific) was to ensure quality control of miRNA concentrations.

### RNA sequencing

Ribo-Zero rRNA removal kits (Illumina) were used to exclude ribosomal RNA molecules (rRNA). RNA sequencing libraries were constructed with the TruSeq Stranded Total RNA Library Prep Kit (Illumina) following the manufacturer’s instructions, and the quality was evaluated by a Bioanalyser 2100 system (Agilent Technologies). Then, single-stranded DNA molecules were clustered and sequenced for 150 cycles on an Illumina HiSeq 2500 sequencing system (Illumina). Finally, paired-end reads were acquired from the Illumina HiSeq sequencer, and quality control was performed with Q30. Removal of 3′ adaptor‐trimming and low‐quality reads was conducted on Cutadapt software (v1.9.3). Alignment of the high-quality clean reads with the human reference genome (UCSC hg19) was performed on hisat2 (v2.0.4) software (http://ccb.jhu.edu/software/hisat2/index.shtml).

Additionally, cuffdiff software (part of cufflinks) was used to determine the FPKM (fragments per kilobase of exon per million fragments mapped) as the expression profiles of mRNAs and lncRNAs with the guidance of the gtf gene annotation file.

### MiRNA sequencing

NEB Next Multiplex Small RNA Library Prep Set for Illumina (New England Biolabs) was performed to construct miRNA sequencing libraries. The quality of the libraries was evaluated by a Bioanalyser 2100 system (Agilent Technologies). Then, the single-stranded DNA molecules from denatured libraries were captured on Illumina flow cells and amplified in situ as clusters. A HiSeq 4000 sequencing system (Illumina) was used for sequencing for 50 cycles according to the manufacturer’s instructions. Cutadapt software (v1.9.3) was used to trim the adaptor sequences from the sequencing data and retain the adaptor-trimmed reads (> = 15 nt). The trimmed reads were merged and used to predict novel miRNAs with miRDeep2 software (v2.0.0.5) [22]. The alignment of the trimmed reads was used both miRBase (http://www.mirbase.org) to obtain known pre-miRNAs and Novoalign software (v3.02.12) (http://www.novocraft.com/main/index.php) for newly predicted pre-miRNAs. The primal expression levels of the miRNA were determined via the numbers of tags on each mature miRNA. The TPM (tag counts per million aligned miRNAs) method was used for the standardization of read counts.

### Analysis of DE lncRNA *cis*-regulatory target mRNAs

The LncRNA Disease database (http://www.cuilab.cn/lncrnadisease) [23] was used to identify overlapping mRNAs of non-intergenic DE lncRNAs, which including bidirectional, exon sense-overlapping, intron sense-overlapping, intronic antisense and natural antisense category DE lncRNAs. *Cis*-regulatory target mRNAs that were involved in HIV infection were determined by the HIV Interaction Database (https://www.ncbi.nlm.nih.gov/genome/viruses/retroviruses/hiv-1/interactions/), [24] LncRNA Disease database and Genecard database (https://www.genecards.org/). Moreover, the neighbouring DE mRNAs were screened within a genomic distance of 300 kb upstream or downstream of each DE lncRNA transcription start and stop site.

### Construction of ceRNA networks

LncLocator [25] and iLoc-LncRNA [26] were used to screen cytoplasmic lncRNAs because only cytoplasmic lncRNAs can function as ceRNAs. Therefore, lncRNAs that were predicted to be located in the nucleus by these two software programs were excluded from analysis. In addition, DE miRNAs that had records in miRbase were screened to construct the ceRNA networks. DE miRNAs that were potential targets of DE lncRNAs were identified in DIANA tools-LncBasev.2 [27]. Notably, the target miRNAs of some DE lncRNAs that were not recorded in DIANA tools-LncBasev.2 were predicted in miRDB databases [28]. Subsequently, DE mRNAs that were potential targets of DE miRNAs were predicted using TargetScan [29] and miRDB databases. CeRNA networks were constructed according to the above analysis and visualized using Cytoscape software (v3.6.0) [30]. Furthermore, some experimentally validated miRNA-mRNAs were queried in the miRTarBase database [31].

### Gene Ontology (GO) annotations and Kyoto Encyclopedia of Genes and Genomes (KEGG) enrichment

GO and KEGG pathway enrichment analyses were performed on the target mRNAs involved in the ceRNA networks using the Database for Annotation, Visualization and Integrated Discovery (DAVID, Version 6.8 Beta) (http://david.abcc.ncifcrf.gov/) [32] online functional annotation tool. GO analysis included three categories: biological process (BP), molecular function (MF), and cellular component (CC). The *p*-value < 0.05 indicated statistically significant enrichment.

### Real‑time quantitative PCR validation

Total RNA was isolated with the miRNeasy Mini Kit (Qiagen). The RNAs was reverse transcribed with the PrimpScript ®RT reagent Kit (TAKARA) for validation of lncRNAs and mRNAs and with the Mir-X™ miRNA First-Strand Synthesis Kit (TAKARA) for validation of miRNAs. TB Green® Premix Ex Taq™ II (TAKARA) was used for RT-qPCR. GAPDH and U6 were employed as endogenous controls for lncRNA/mRNA RT-qPCR and miRNA RT-qPCR, respectively. The relative expression of lncRNAs, mRNAs and miRNAs was calculated by the 2^−△△Ct^ method.

### Statistical analysis

A nonparametric Mann–Whitney U test was performed to investigate differentially expressed genes. Data were analysed, and graphs were created using GraphPad Prism v8.0. Two-tailed *p*-value of less than 0.05 was considered statistically significant.

## Results

### Identification of differentially expressed lncRNAs, mRNAs and miRNAs between HAART‑naive EHI patients and HCs

**Fig. 1:**
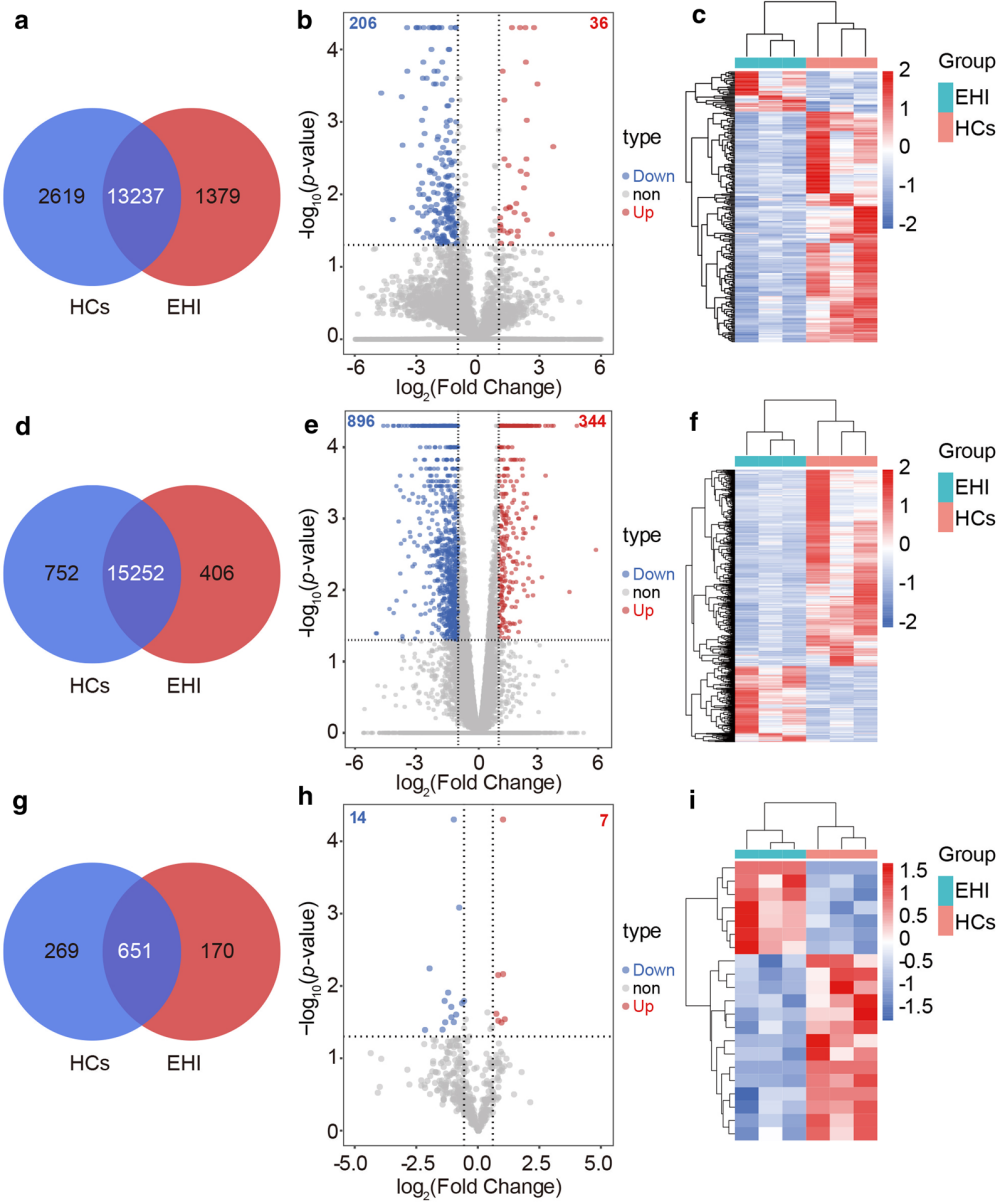
Expression profile of lncRNAs, mRNAs and miRNAs. Venn diagrams, volcano plots and heatmaps of lncRNAs (**a**–**c**), mRNAs (**d**–**f**) and miRNAs (**g**–**i**) are shown. Venn diagrams show the common and specific lncRNAs, mRNAs and miRNAs in HAART‑naive EHI patients and HC groups. Volcano plots show upregulated (red) and downregulated (blue) DE lncRNAs (with *p* < 0.05 and fold change ≥ 2), DE mRNAs (with *p* < 0.05 and fold change ≥ 2) and DE miRNAs (with *p* < 0.05 and fold change ≥ 1.5). Heatmaps show hierarchical clustering of DE lncRNAs, DE mRNAs and DE miRNAs

A total of 17,235 lncRNAs were detected (Additional file 2: Table S2). Overall, 2619 lncRNAs were only identified in the HC group, 1379 lncRNAs were only identified in the EHI patients, and 13,237 lncRNAs were identified in both groups (Fig. 1a). Thirty-six upregulated lncRNAs and 206 downregulated lncRNAs were differentially expressed (*p* < 0.05, fold change ≥ 2) (Fig. 1b and c). For mRNAs, a total of 16,410 mRNAs were detected (Additional file 3: Table S3). 752 mRNAs were identified only in the HC group, 406 mRNAs were identified only in the EHI patients, and 15,252 mRNAs were identified in both groups (Fig. 1d). A total of 344 upregulated mRNAs and 896 downregulated mRNAs were differentially expressed (*p* < 0.05, fold change ≥ 2) (Fig. 1e and f). Finally, a total of 1090 mature miRNAs were obtained via miRNA-Seq (Additional file 4: Table S4). Among these miRNAs, 269 miRNAs were identified only in the HC group, 170 miRNAs were identified only in the EHI patients, and 651 miRNAs were detected in both groups (Fig. 1g). Twenty-one DE miRNAs, including 7 significantly upregulated miRNAs and 14 significantly downregulated miRNAs (*p* < 0.05, fold change ≥ 1.5), were identified from 691 known miRNAs recorded in miRbase (Fig. 1h and i).

### Features of DE lncRNAs expression profiling

**Fig. 2:**
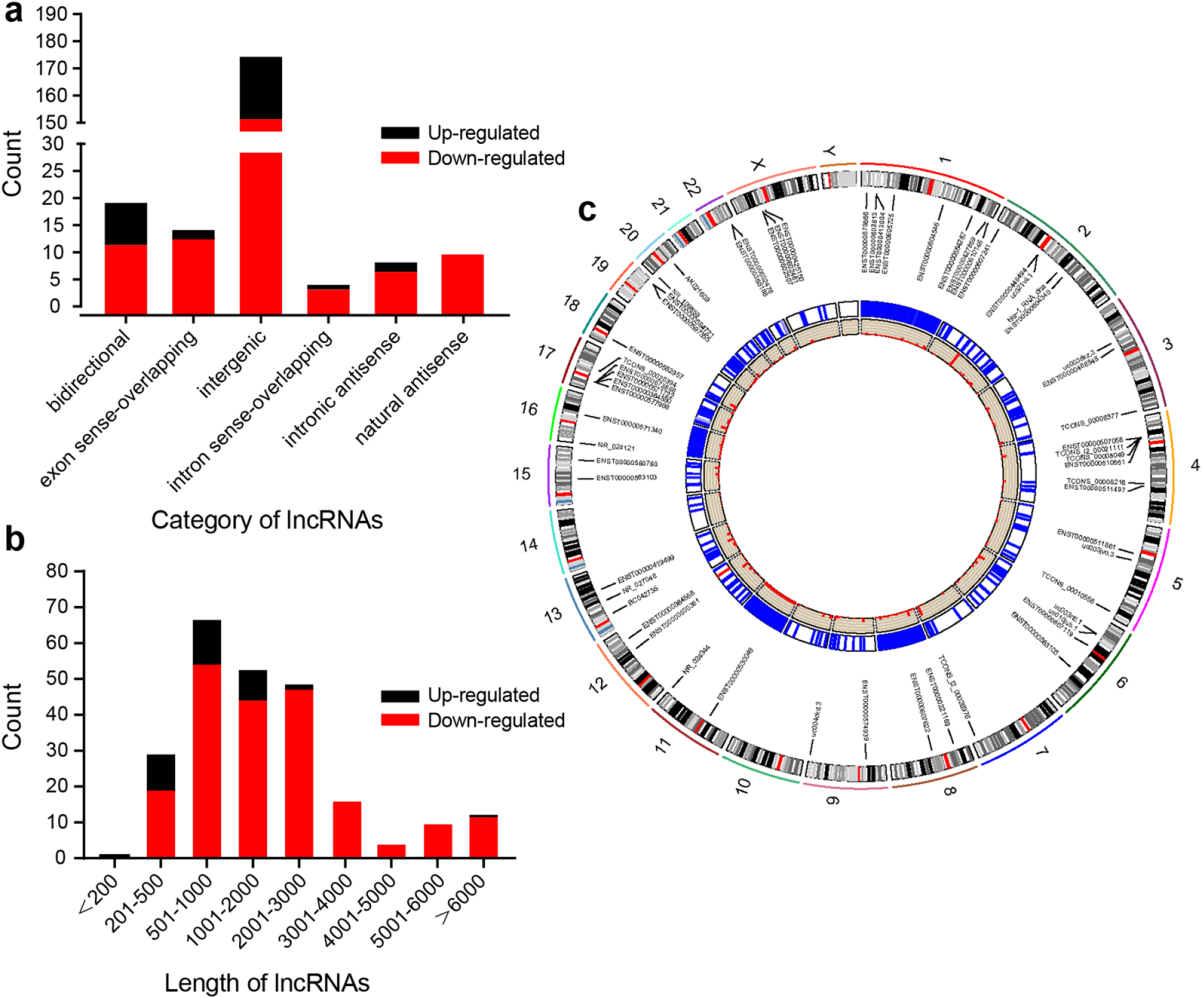
Profiling and characteristics of DE lncRNAs. **a** Category distribution of DE lncRNAs. **b** Length distribution of DE lncRNAs. **c** Circos plot shows distribution of DE lncRNAs corresponding to human chromosomes. The outermost layer is a chromosome map of the human genome. White and black bands represent chromosome cytobands, while red bands represent centromeres. The top 30 significantly upregulated DE lncRNAs and top 30 significantly downregulated DE lncRNAs are shown in the second layer. The third heatmap layer shows the expression level of all DE lncRNAs. Red represents a high level of expression, and blue represents a low level of expression. The innermost histogram layer shows the fold change of all DE lncRNAs

We further characterized the expression profiling of 242 DE lncRNAs. First, DE lncRNAs were categorized into six groups based on the chromosomal position relationship of the associated coding genes, namely, bidirectional, exon sense-overlapping, intergenic, intron sense-overlapping, intronic antisense, and natural antisense, with constituent ratios of 8.68% (21/242), 6.61% (16/242), 73.55% (178/242), 2.48% (6/242), 4.13% (10/242) and 4.55% (11/242), respectively (Fig. 2a). Second, the length range of lncRNAs was from 131 nt to over 6000 nt. The majority of the DE lncRNAs were distributed in 3 intervals: 501–1000, 1001–2000 and 2001–3000 (Fig. 2b). Third, DE lncRNAs were located on 22 autosomes and the X chromosome (Fig. 2c). A higher number of DE lncRNAs were located on chromosomes 1, 8, 11, and 16. Furthermore, expression levels of most DE lncRNAs were low.

### Exploration of the potential roles of lncRNAs through *cis*-regulatory mechanisms

LncRNAs regulate the expression of nearby protein-coding genes in a *cis*-regulatory fashion. First, we investigated non-intergenic lncRNAs that overlapped with mRNAs. Forty-four lncRNAs were predicted to overlap with 41 *cis*-regulatory target mRNAs (Additional file 5: Table S5). Among these target mRNAs, 6 mRNAs were differentially expressed and might be targets of 6 DE lncRNAs. These 6 lncRNA-mRNA pairs were all consistently regulated, including 3 pairs up and 3 pairs down (Additional file 5: Table S5). Furthermore, 4 DE mRNAs that were possibly involved in HIV infection exhibited the same regulatory direction as 4 lncRNAs. Specifically, *FCAR* and *HIST1H2BJ* overlapped with 2 exon sense-overlapping lncRNAs, and *CYB5R3* and *AP3M2* overlapped with 2 bidirectional lncRNAs (Table 1).

**Table 1:** Several potential *cis*-regulatory DE lncRNA-mRNA pairs

Category^a^	lncRNA	Log2 (fold change)	*P* value	mRNA	Log2 (fold change)	*P* value
Exon sense-overlapping	ENST00000594721^b^	1.264	0.003	FCAR	1.259	< 0.001
Exon sense-overlapping	uc003niu.1^c^	1.061	0.021	HIST1H2BJ	1.350	0.001
Bidirectional	ENST00000602478^b^	2.878	< 0.001	CYB5R3	1.128	0.011
Bidirectional	ENST00000564481^b^	− 1.533	0.015	AP3M2	− 1.212	0.002
Bidirectional	ENST00000566814^b^	− 1.444	0.013	SEZ6L	− 1.288	0.010
Intergenic	ENST00000456129^b^	− 1.721	0.002	SEZ6L	− 1.288	0.010
Intergenic	ENST00000423278^b^	− 1.416	< 0.001	SEZ6L	− 1.288	0.010
Intergenic	ENST00000430080^b^	− 1.392	0.010	SEZ6L	− 1.288	0.010
Intergenic	ENST00000364880^b^	3.576	0.036	GRAP	− 1.276	< 0.001
Intergenic	ENST00000571722^b^	2.358	0.001	GRAP	− 1.276	< 0.001
Intergenic	ENST00000577988^b^	2.376	0.023	GRAP	− 1.276	< 0.001
Intergenic	ENST00000492960^b^	− 2.152	< 0.001	ZAP70	− 1.275	< 0.001
Intergenic	TCONS_00006930^d^	− 1.030	0.010	BTLA	− 1.209	0.007

^a^The chromosomal position relationship of lncRNAs and associated coding genes

The source database of lncRNAs is ^b^Ensembl ^c^UCSC_knowngene ^d^TCONS

Second, we predicted the potential target mRNAs nearby DE lncRNAs. We found 21 lncRNA-mRNA pairs from 36 upregulated lncRNAs and 86 lncRNA-mRNA pairs from 206 downregulated lncRNAs (Additional file 5: Table S5). Among these lncRNA-mRNA pairs, 18 pairs were inversely regulated. Three upregulated intergenic lncRNAs (ENST00000364880, ENST00000571722 and ENST00000577988) were predicted to target the downregulated mRNA *GRAP* (Table 1). Four downregulated lncRNAs (ENST00000456129, ENST00000566814, ENST00000423278 and ENST00000430080) were predicted to target the downregulated mRNA* SEZ6L* (Table 1). In addition, the downregulated intergenic lncRNAs ENST00000492960 and TCONS_00006930 were predicted to target the downregulated mRNAs *ZAP70* and *BTLA,* respectively, which were associated with T-cell activation and HIV-1 replication in HIV infection (Table 1).

### Exploration of the potential roles of lncRNAs through ceRNA networks

**Fig. 3:**
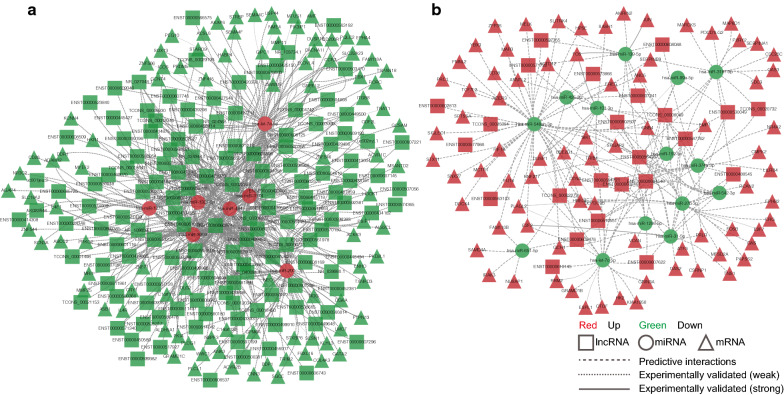
CeRNA network analysis of lncRNAs, miRNAs and mRNAs. **a** Networks of downregulated lncRNAs, upregulated miRNAs and downregulated mRNAs. **b** Networks of upregulated lncRNAs, downregulated miRNAs and upregulated mRNAs. Strong (solid lines) and weak (dotted lines) evidence of experimentally validated interactions and predictive interactions (dashed line) between lncRNAs (squares), miRNAs (circles) and mRNAs (triangles) are shown. Red and green represent upregulated and downregulated genes, respectively

To further elucidate the potential interactions of DE lncRNAs, miRNAs and mRNAs involved in EHI patients, lncRNA-miRNA-mRNA networks were constructed. A total of 160 DE lncRNAs, 21 DE miRNAs and 175 DE mRNAs were selected to construct 2 ceRNA networks: one contained 136 downregulated lncRNAs, 7 upregulated miRNAs and 97 downregulated mRNAs, and the other contained 24 upregulated lncRNAs, 14 downregulated miRNAs and 78 upregulated mRNAs (Fig. 3). We found that most lncRNAs shared miRNA response elements (MREs) of multiple miRNAs, and most miRNAs regulated multiple mRNAs. For example, 88 downregulated lncRNAs might function as ceRNAs of the upregulated hsa-miR-484 targeting 14 downregulated mRNAs (Fig. 3a). Seventeen upregulated lncRNAs might share MREs of the downregulated hsa-miR-548ah-3p with 37 upregulated mRNAs (Fig. 3b). Three lncRNAs, ENST00000504409, TCONS_00006930 and NR_024368, were predicted to have MRE binding sites for all 7 upregulated miRNAs (Fig. 3a). Furthermore, lncRNA ENST00000564287 was predicted to share MREs of 13 downregulated miRNAs (Fig. 3b). Moreover, 31 experimentally validated miRNA-mRNA interactions were found in the ceRNA networks and might be related to 134 DE lncRNAs (Additional file 6: Table S6).

### GO and KEGG enrichment analysis of lncRNA-associated mRNAs in ceRNA networks

**Fig. 4:**
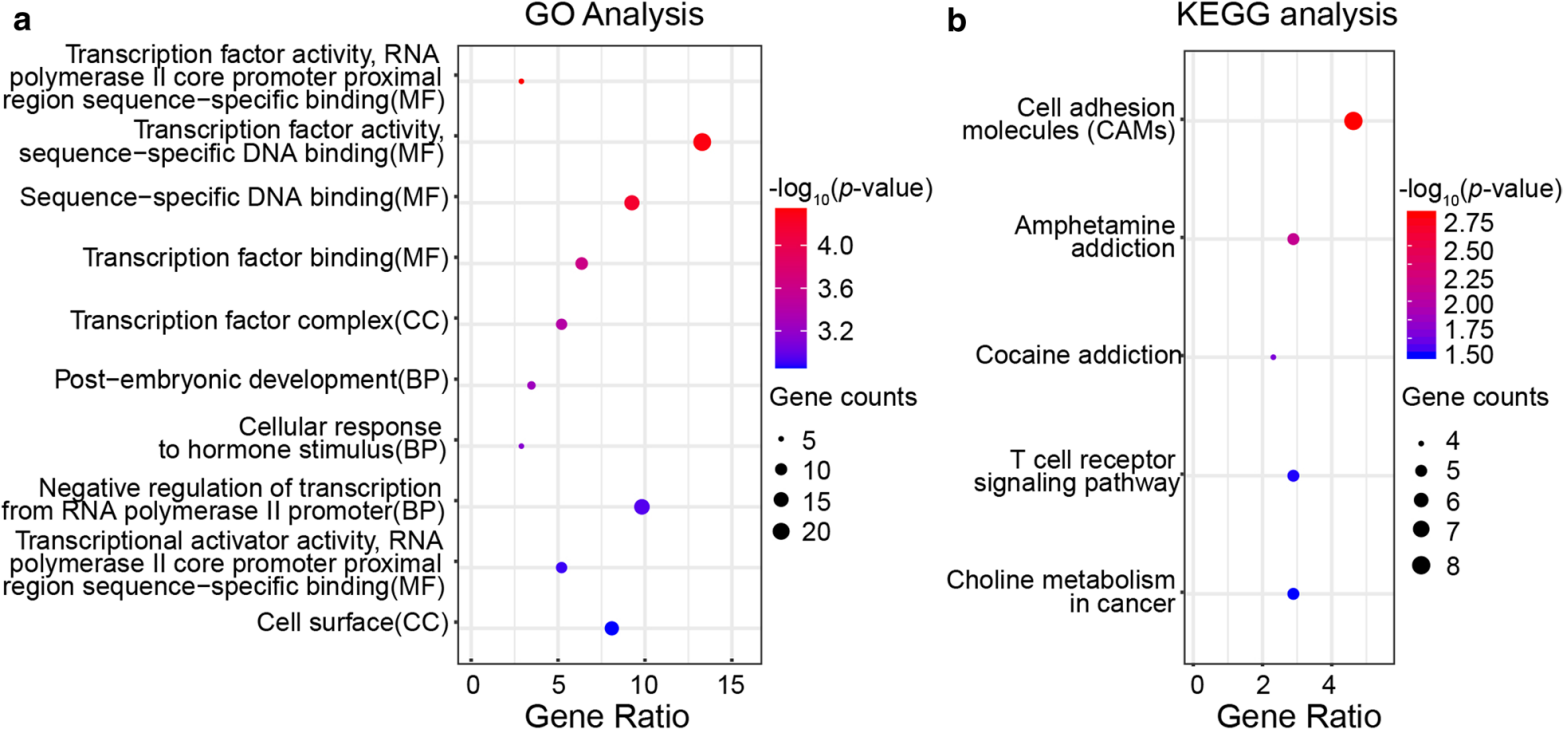
GO and KEGG pathway analysis of DE lncRNA-targeted mRNAs in ceRNA networks. **a** The top 10 enriched GO terms of lncRNA-targeted mRNAs, including biological process (BP), cellular component (CC) and molecular function (MF). **b** Enriched KEGG pathways of lncRNA-targeted mRNAs. *p* < 0.05 was defined as the threshold for the enrichment analysis

**Fig. 5:**
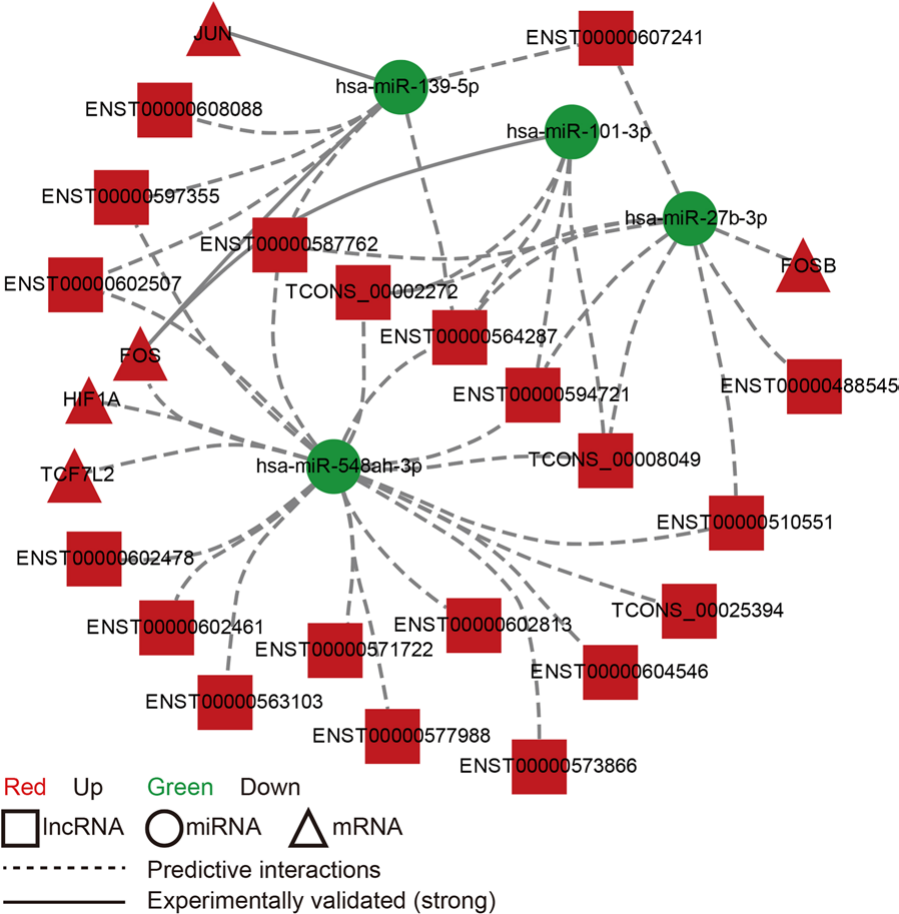
LncRNA-associated ceRNA networks might be involved in HIV-1 replication and immune activation during EHI. Strong (solid lines) evidence of experimentally validated interactions and predictive interactions (dashed lines) between lncRNAs (squares), miRNAs (circles) and mRNAs (triangles) are shown. Red and green represent upregulated and downregulated genes, respectively

To explore the potential roles of lncRNAs in lncRNA-associated ceRNA networks, 175 DE mRNAs involved in the ceRNA networks were selected for GO and KEGG enrichment analysis (Additional file 7: Table S7). The top 5 GO terms were transcription factor activity, RNA polymerase II core promoter proximal region sequence-specific binding (GO: 0000982), transcription factor activity, sequence-specific DNA binding (GO: 0003700), sequence-specific DNA binding (GO: 0043565), transcription factor binding (GO: 0008134), and transcription factor complex (GO: 0005667) in ascending order of *p*-values (Fig. 4a). Five upregulated mRNAs, *HIF1A*, *TCF7L2*, *FOS, FOSB and JUN* occurred in several GO terms. Seventeen DE lncRNAs were predicted to share the MRE with hsa-miR-548ah-3p and in turn regulate expression of *HIF1A* and *TCF7L2,* which were reported to impact HIV-1 replication (Fig. 5).

In addition, 5 KEGG pathways were identified among the DE mRNAs involved in the lncRNA-associated ceRNA networks (Fig. 4b). We observed that 5 genes, *JUN, PLCG1, ICOS, FOS* and *CD28,* were enriched in the T cell receptor signalling pathway (hsa04660). Among these mRNAs, the proteins encoded by the *FOS, JUN* and *FOSB* genes are the main subunits of activating protein 1 (AP-1), a vital transcription factor in HIV replication and the immune response. Four DE miRNAs, miR-101-3p, miR-139-5p, miR-548ah-3p and miR-27b-3p, were found to target the above 3 mRNAs. In addition, a total of 20 DE lncRNAs were predicted to share MREs with *FOS, JUN* and *FOSB* and might regulate AP-1 as ceRNAs (Fig. 5). Note that hsa-miR-101-3p/*FOS* and hsa-miR-139-5p/*FOS*/*JUN* in ceRNA networks were experimentally validated (Additional file 6: Table S6). Therefore, these results provide reliable evidence for further exploring the functions of lncRNAs in EHI.

### Real‑time quantitative PCR validation

**Fig. 6:**
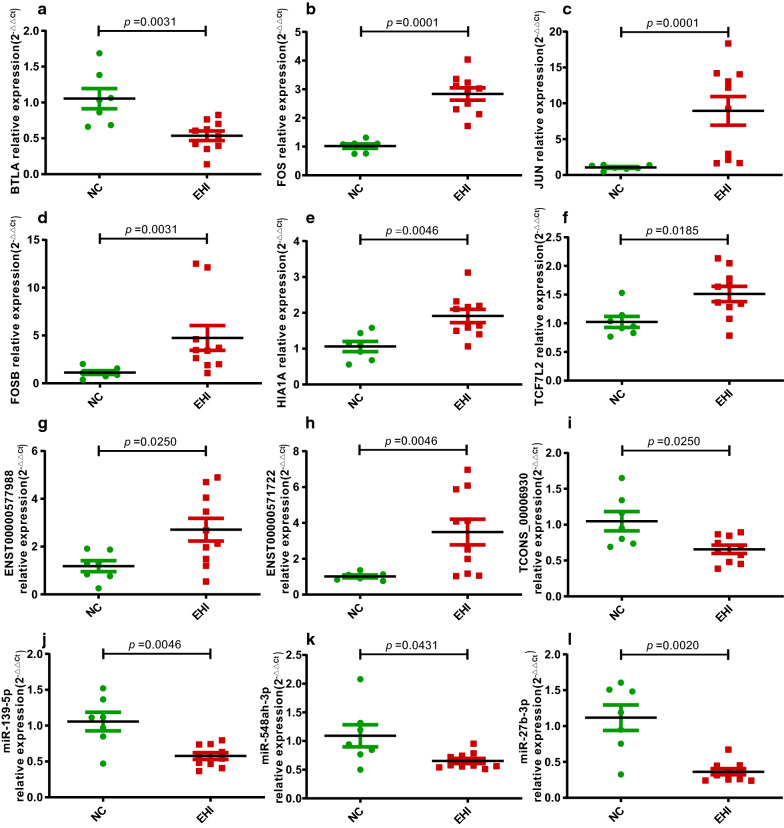
Validations of selected DE RNA transcriptions via RT-qPCR. Six DE mRNAs (**a**–**f**), 3 DE lncRNAs (**g**–**i**) and 3 DE miRNAs (**j**–**l**) were validated

The lncRNAs, miRNAs and mRNAs mentioned above, which were predicted to play important roles during early HIV infection, were validated by RT-qPCR. A total of 15 DE RNA transcripts were validated, including 6 mRNAs, 5 lncRNAs, and 4 miRNAs. The results of 12 (80%, 12/15) RNA transcriptions were consistent with the sequencing data (*p* < 0.05) (Fig. 6), except for lncRNA ENST00000364880, ENST00000492960 and miRNA miR-101-3p (data not shown).

## Discussion

In this study, we analysed lncRNA, miRNA and mRNA profiles and explored the potential functions of lncRNAs during EHI. A total of 1240 mRNAs, 242 lncRNAs and 21 known miRNAs were identified as differentially expressed during EHI. We found that several lncRNAs might regulate *ZAP70*, *BTLA* and *GRAP* in a *cis*-regulatory fashion. Some lncRNAs were predicted to act as ceRNAs to regulate expression of *HIF1A*, *TCF7L2*, *FOS*, *FOSB* and *JUN*. Some target mRNAs here found regulated directly or indirectly by lncRNAs have been previously reported to impact HIV replication and immune activation. Therefore, our findings may provide new ideas for further study to explore the roles of lncRNAs during EHI and help to understand the interactions between the host and HIV virus.

LncRNAs are considered to be lower expressed compared to protein coding mRNAs, and the expression of lncRNAs is spatiotemporal, and tissue-and cell-specific [33–36]. Dysregulated lncRNAs have been found to play important roles in various diseases [37, 38], including HIV infection[39]. Several lncRNAs have been found to impact HIV-1 viral replication, the establishment of latency and the HIV-associated immune response [39]. For instance, lncRNA MALAT1 (metastasis-associated lung adenocarcinoma transcript 1) [40], uc002yug.2 [41] and HEAL (HIV-1-enhanced lncRNA) [42] were reported to activate HIV transcription, while NRON (noncoding repressor of nuclear factor of activated T cells) [43, 44], NEAT1 (nuclear paraspeckle assembly transcript 1) [45, 46] and AK130181 [47] were found to repress HIV transcription. In addition, LINC00173 was shown to regulate the cytokine secretion of T cells and further affect HIV-associated immune responses [48]. However, the functions of lncRNAs during EHI have not been reported thus far. In this study, we predicted that some lncRNAs might play important roles in HIV-1 replication and immune activation during EHI.

In this study, we speculated that some lncRNAs might regulate genes in a *cis*-regulatory fashion during EHI. For example, lncRNA TCONS_00006930, which was identified as a cell cycle‑associated lncRNA in endometriosis [49] was downregulated in EHI patients and may regulate the expression of *BTLA*. BTLA and ZAP70 have been found to regulate T cell activation during HIV infection [50–52]. In addition, ZAP-70 is required for efficient cell to cell HIV transmission and for the formation of virological synapses that promote HIV replication [53]. In our study, we predicted that lncRNA ENST00000492960 might regulate the expression of *ZAP-70* and in turn control HIV-1 replication and impact T-cell activation. Furthermore, 3 lncRNAs were predicted to regulate the expression of *GRAP*. GRAP plays a negative regulatory role in lymphocyte proliferation induced by T-cell receptor, interleukin-2 production and c-fos induction [54]. These 3 lncRNAs might belong to the small nucleolar RNA (snoRNA)-ended lncRNAs (sno-lncRNAs) family. It has been reported that sno-lncRNAs may play important regulatory roles in RNA splicing and ribosomal RNA (rRNA) biogenesis [55–57]. However, the potential regulatory relationships between the 3 lncRNAs and *GRAP* need further investigation.

LncRNAs can act as ceRNAs or “miRNA sponges”, for example, lncRNA GAS5 acts on miR-873, resulting in the suppression of HIV replication [58]. In our study, we found that 20 lncRNAs might regulate the expression of *FOS, JUN*, *FOSB, HIF1A* and *TCF7L2* mRNAs as ceRNAs and then affect HIV replication and immune activation during EHI. *FOS*, *JUN* and *FOSB* code the common protein components (c-Fos, c-Jun and FosB) of activating protein 1 (AP-1) and they are strong activators of AP-1 [59]. It has been reported that HIV gp120 induces endogenous c-fos and c-jun expression to enhance the function of AP-1 [60]. AP-1 performs vital functions in T-cell activation, Th differentiation, T-cell anergy and exhaustion [61–63], affecting the establishment of HIV viral latency [64]. In addition, several studies found that c-FOS was conducive to HIV-1 replication [65], while FosB reduced the promoter activity of the HIV long terminal repeat (LTR) [66]. Interestingly, HIF1A also can promotes HIV replication in CD4^+^ T cells [67] and the HIV-1 accessory protein Vpr was shown to activate the LTR in an HIF1A-dependent manner in human microglial cells [68, 69]. However, TCF7L2 (TCF-4) can repress HIV replication in multiple cell types [70–73]. In astrocytes, TCF-4 was reported to interact with β-catenin and SMAR1 and then bind to LTR to decrease basal HIV transcription [72], and TCF-4 also decreases Sp-1-mediated transcription of the HIV promotor [73]. Taken together, our findings revealed that lncRNAs may act as ceRNAs to impact HIV-1 replication and the host viral immune response during EHI.

In our study, 19 lncRNA/miR-101-3p/miR-139-5p/miR-548ah-3p/*FOS*, 6 lncRNA/miR-139-5p/*JUN*, 8 lncRNA/miR-27b-3p/*FOSB* and 17 lncRNA/miR-548ah-3p/*HIF1A/ TCF7L2* were listed in the ceRNA networks. Among these 20 DE lncRNAs, lncRNA ENST00000602461 and ENST00000602507 were different transcriptions of lncRNA MIR222HG (Lnc-Ang362) which is the host transcript for miR-221 and miR-222. LncRNA MIR222HG were reported to mediate the proliferation of vascular smooth muscle cells [74] and facilitate the development of castration-resistant prostate cancer [75]. In addition, lncRNA ENST00000602478, ENST00000488545 and ENST00000587762 were differentially expressed in coxsackievirus A16 (CVA16) infection, human gastric cancer and astrocytoma, respectively [76–78]. These lncRNAs may be new targets for the diagnosis and treatment. However, the functions of these 20 lncRNAs in HIV-1 infection were not reported previously and the mechanisms need to be further studied.

However, our study has several limitations. First, RNA-seq and miRNA-seq were performed on total PBMCs that contain multiple cell types; thus, these results represent the most prominent or common biological processes during EHI. Further studies are needed to identify the roles of lncRNAs in specialized cell types. Second, we predicted the potential roles of lncRNAs during EHI by bioinformatics analysis. Although some experimentally validated gene regulatory networks appeared in our analysis, it is still necessary to validate these results with related experiments. Third, the sample size is small because RNA samples from HAART-naive EHI patients are difficult to collect. A larger sample size is needed to verify our results.

## Conclusions

For the first time, our study identified a lncRNA profile and predicted several lncRNAs that might play a role in HIV-1 replication and immune activation during EHI. These novel findings contribute to our understanding of the pathogenesis of HIV infection during EHI and provide new insights into antiviral therapy and vaccine development.

## Supplementary Information

**Additional file 1: Table S1.** Clinical characteristics of participants

**Additional file 2: Table S2.** LncRNA expression profiling.

**Additional file 3: Table S3.** mRNA expression profiling.

**Additional file 4: Table S4.** miRNA expression profiling.

**Additional file 5: Table S5.** Predictive *cis*-target mRNAs of differentially expressed lncRNAs.

**Additional file 6: Table S6. **Experimentally validated miRNA-mRNA interactions from miRTarBase in ceRNA network.

**Additional file 7: Table S7.** Enrichment analyses of target mRNAs in ceRNA neworks.

## Data Availability

All relevant data and materials during this study are included in this published article and its supplementary information files.

## Abbreviations

HIV-1: Human immunodeficiency virus 1
AIDS: Acquired immunodeficiency syndrome
EHI: Early HIV infection
lncRNA: Long noncoding RNA
ceRNA: Competing endogenous RNA
miRNA: MicroRNA
PBMC: Peripheral blood mononuclear cell
HC: Healthy control
RNA-Seq: RNA sequencing
miRNA-Seq: MiRNA sequencing
GO: Gene Ontology
KEGG: Kyoto Encyclopedia of Genes and Genomes
MRE: MiR response element
BTLA: B and T lymphocyte attenuator
ZAP70: Zeta chain of T cell receptor-associated protein kinase 70
GRAP: GRB2-related adaptor protein
HIF1A: Hypoxia-inducible factor-1A
TCF7L2: Transcription factor 7-like 2
AP-1: Activating protein 1
RT-qPCR: Real-time quantitative PCR
